# Premature aging and metabolic diseases: the impact of telomere attrition

**DOI:** 10.3389/fragi.2025.1541127

**Published:** 2025-03-31

**Authors:** Sandhya Jinesh, Burçin Özüpek, Prerana Aditi

**Affiliations:** ^1^ CVS Health, Trumbull, CT, United States; ^2^ Independent Researcher, Wylie, TX, United States; ^3^ Department of Medical Biochemistry, Faculty of Allied Health Sciences, Mahayogi Gorakhnath University, Gorakhpur, Uttar Pradesh, India

**Keywords:** aging, premature aging, telomere, telomerase, metabolic diseases

## Abstract

Driven by genetic and environmental factors, aging is a physiological process responsible for age-related degenerative changes in the body, cognitive decline, and impaired overall wellbeing. Notably, premature aging as well as the emergence of progeroid syndromes have posed concerns regarding chronic health conditions and comorbidities in the aging population. Accelerated telomere attrition is also implicated in metabolic dysfunction and the development of metabolic disorders. Impaired metabolic homeostasis arises secondary to age-related increases in the synthesis of free radicals, decreased oxidative capacity, impaired antioxidant defense, and disrupted energy metabolism. In particular, several cellular and molecular mechanisms of aging have been identified to decipher the influence of premature aging on metabolic diseases. These include defective DNA repair, telomere attrition, epigenetic alterations, and dysregulation of nutrient-sensing pathways. The role of telomere attrition premature aging in the pathogenesis of metabolic diseases has been largely attributed to pro-inflammatory states that promote telomere shortening, genetic mutations in the telomerase reverse transcriptase, epigenetic alteration, oxidative stress, and mitochondrial dysfunctions. Nonetheless, the therapeutic interventions focus on restoring the length of telomeres and may include treatment approaches to restore telomerase enzyme activity, promote alternative lengthening of telomeres, counter oxidative stress, and decrease the concentration of pro-inflammatory cytokines. Given the significance and robust potential of delaying telomere attrition in age-related metabolic diseases, this review aimed to explore the molecular and cellular mechanisms of aging underlying premature telomere attrition and metabolic diseases, assimilating evidence from both human and animal studies.

## 1 Introduction

Aging is defined as a physiological phenomenon driven by genetic and biological processes, which are related to the lifespan of an individual and are associated with all age-related pathologies (Li et al., 2021). The aging process increases the susceptibility of individuals to factors leading to death as they grow older. Aging is a complex multifactorial phenomenon that involves the simultaneous interaction between various factors at different levels of functional organization. The role of genetic and environmental factors is represented by the heterogenous aging phenotype across different individuals, hence, these factors influence the lifespan of an individual via the process of aging (Jayanthi et al., 2010). With the deterioration of physiological functions critical to the survival and fertility of humans, the process of aging is known to relate to the notion of natural selection (Gilbert, 2000). According to gerontologists and biologists, it is implicated that the process of aging starts at birth, however, age-related structural and functional alterations tend to occur after humans achieve sexual maturity (Butler et al., 1987). Pathological aging is an illness that affects older people, such as neurological diseases, whereas normal aging is a natural process that involves slow changes in the body.

In the context of age-related degeneration, the degenerative changes are not as significantly impactful as the ability of individuals to respond and adapt to these degenerative changes. The perspective of healthy aging can be described as the development of positive environments and the capacity of individuals to meet their values (Kyriazis, 2020). In addition to structural and functional limitations, there are correlating cognitive changes that occur with normal aging. Moreover, each of the cognitive domains demonstrates a decline with age. The more noticeable changes in cognitive decline include a reduction performance of complex attentional tasks and selective attention. Moreover, there is a consistent decrease in new learning abilities and prospective memory whereas procedural memories are preserved with aging (Murman, 2015). Lifetime cumulative adversity plays a pivotal role in exacerbating age-related dysfunction across physical, cognitive, and mental health markers (Shrira, 2014).

According to the World Health Organization, every sixth individual will be of age 60 years or above by the year 2030, across the globe. The pool of individuals aged 60 years and above is estimated to grow to 1.4 billion by 2030 and 2.1 million by 2050. Moreover, the number of individuals aged 80 years and above is expected to reach up to 426 million by the year 2050 (Noto, 2023). The increase in the older population in both developed and developing countries has raised concerns regarding the overall wellbeing and quality of life of the elderly (Das and Bhattacharyya, 2022). The substantial rise in the aging population has contributed to the global burden of diseases. The proportion of this burden arising from disorders in the elderly is highest in developed countries, the disability-adjusted life years, however, are higher in developing countries (Prince et al., 2015).

While aging is a physiological phenomenon, it serves as a driving factor for several age-related disorders. The underlying aging mechanisms may include genomic instability, shortening of the telomere, epigenetic alterations, loss of proteostasis, deregulated nutrient sensing, stem cell exhaustion, mitochondrial dysfunction, altered intercellular communication, and cellular senescence (Li et al., 2021). The age-related increase in the risk of degenerative disease is linked to age-related deterioration in cellular biological functions. These biological functions are related to nutrient sensing, energy metabolism, and cellular proliferation (Pyo et al., 2020). Notably, genetic risk factors play an important role in influencing the risk of development of diseases at different age intervals. This is mediated by the effect of genetic factors on the molecular, tissue, and cellular phenotypes, which can be influenced by acute and chronic environmental exposures. There is, however, underestimation and overestimation of the absolute risk for younger and older individuals with a greater polygenic burden of diseases, respectively (Jiang et al., 2021) (Table 1).

**TABLE 1:** Genes involved in metabolic disorders.

Study Type (Animal/Clinical)	Gene list	Population	Type 2 diabetes (T2D)
Clinical (Sethi et al., 2020)	*OBFC1* (rs9419958 variant) *TERF2* (rs4783704 variant) *TERC/LRRC31* (rs16847897 variant) *TERC/MYNN* (rs10936599 variant) *CSNK2A2* (rs74019828 variant)	Northwest Indian population	These variants of telomere maintenance genes were found to be involved in the pathogenesis of T2DM in the Northwest Indian population. Of the five variants, only two, rs10936599 of the *TERC/MYNN* gene and rs74019828 of the *CSNK2A2* gene, were significantly associated with T2DM in the females.
Clinical (Alwan and Research, 2023)	*TERT* (rs2736100 variant)	Iraqi	Homozygous genotypes of *TERT* gene variants rs2736100 were found to be associated with increased susceptibility to T2DM, with comparable effects across both genders for gene polymorphisms rs2736100 variant A < C.
Obesity			
Animal (Bloom et al., 2020)	*POT1b* *TNFα* *MCP1 p53*	Mice	The gene expression of POT1b was found to be increased in aged mice regardless of diet, indicating that advancing age drives the increase in POT1b expression. The mRNA expression of MCP1 and TNFα genes was significantly associated with body weight, and the expression of these genes was associated with alteration in the expression of telomere-related genes.
Animal (Glasstetter et al., 2023)	*TERT* *CDKN2C* *MAPK10*	Female domestic pigs	The study demonstrated the association of obesity and dyslipidemia with dysregulation of DNA hydroxy methylation of genes related to apoptosis and senescence, as studied in animal and human mesenchymal stem cells. The epigenetic 5 hmc marker levels of CDKN2C and TERT protein expression were raised compared to 5 hmc levels of the MAPK10 gene in obese mesenchymal stem cells.
Neurological disorders			
Animal (Mojiri et al., 2021)	*EndoMT* *Periostin* *Snail* *TGF-ß* *SIRT1*	Progeria mice	The treatment of the Hutchinson-Gilford syndrome animal model with human-derived TERT is associated with a decrease in the expression of progerin and an increase in the expression of the SIRT1 gene, with the latter involved in the maintenance of telomeres and reduction in DNA damage signaling at telomeres. Hutchinson-Gilford syndrome cells exhibit a reduction in the co-localization of SIRT1 and the telomere, which is revered by treatment with TERT. The treatment has also been demonstrated to reverse the increase in genes linked to endothelial-to-mesenchymal transition (EndoMT) including Periostin, Snail, and TGF-ß.
Liver Cirrhosis			
Clinical (Faingelernt et al., 2022)	*TERT* *TERC*	Two healthy and three affected patients	Patients with telomere diseases may develop early-onset liver failure including end-stage liver disease.
Chronic Kidney Disease			
Animal (Saraswati et al., 2021)	*Trf1*	Mouse	The deletion of the gene encoding the shelterin telomere protective complex Trf1 protein is associated with short telomeres, which sensitize the kidneys to undergo fibrosis. Trf1 deletion may also trigger the activation of epithelial-to-mesenchymal transition, leading to pathological age-associated fibrosis.
Clinical (Su et al., 2024)	*TERT (rs2735940 and rs4635969 variants)*	Chinese	TERT gene variants rs2735940 and rs4635969 are correlated with an increased risk of chronic kidney disease, with rs2735940 variant homozygous genotype associated with increased microalbuminuria in chronic kidney disease patients.

The age-related onset of neurodegenerative diseases is linked to increased disability and decreased cognitive and motor functions in the elderly population. Neurodegeneration is a characteristic feature of Parkinson’s disease, amyotrophic lateral sclerosis, and Alzheimer’s disease, however, neurodegenerative changes are also evident in traumatic brain injury, injury to the spinal cord, ischemia, multiple sclerosis, and stroke. The process of aging contributes to the progression and exacerbation of neurodegeneration and is also associated with comorbidities in the elderly (Mayne et al., 2020). In addition to neurodegenerative disease, aging also mediates the development of cardiovascular disorders, immune system disorders, cancer, and musculoskeletal pathologies (Li et al., 2021). Age-related metabolic disorders including obesity and diabetes are associated with several underlying mechanisms including mitochondrial dysfunction. An increase in the production of free radicals, related to biological aging, decreases oxidative capacity, antioxidant defense, and energy metabolism, which subsequently leads to impaired metabolic homeostasis (Bhatti et al., 2017).

Moreover, the global population is undergoing a significant demographic transition characterized by an increasing proportion of older adults, primarily driven by declining birth rates and enhanced longevity (Wan Ahmad et al., 2015; Kuzanashvili and Shioshvili; Amad et al., 2015). The reduction in fertility rates is reshaping family structures and contributing to diminished population growth, while improvements in healthcare and living conditions are extending life expectancy and decreasing mortality rates (Shioshvili, 2024). Although this phenomenon is observed globally, developed countries exhibit a relatively higher proportion of older individuals within their population structures (Wan Ahmad et al., 2015). The aging population presents distinctive social and economic challenges, particularly concerning healthcare, pension systems, and labor markets (Shioshvili, 2024).

Premature aging can be defined as the acceleration of normal aging processes and related clinical features. Premature aging syndromes or progeroid syndromes are associated with impaired replication and repair of the DNA and disrupted nuclear lamina (Foo et al., 2019). Moreover, there are various chronic health conditions and comorbidities that are associated with premature aging, the characteristic features of which include premature vascular aging and diseases vasculature, muscle wasting, osteoporosis, and frailty (Tanriover et al., 2023). Premature aging is a consequence of mutations in the genes encoding for proteins associated with genomic stability, mitochondrial functions, architecture of the nuclear lamina, and various other cellular and biological processes (Coppedè, 2021).

## 2 Cellular and molecular mechanisms of aging

### 2.1 DNA damage

The integrity of genetic information and genome maintenance is critical to the fidelity of replication and transcription processes; however, it is frequently challenged by genotoxic events such as deamination, oxidation, and alkylation of the bases. The subsequent DNA damage interferes with the replication and transcription of genetic material. DNA damage activates the DNA damage response, activating the DNA repair pathways and mediating chromatin remodeling. Appropriate and coordinated control of DNA repair and transcription processes allows the protection of cellular functions (Bordin et al., 2021). Robust DNA repair mechanisms promote overall cellular survival and stabilization of metabolic homeostasis. The major DNA repair pathways are as follows: (a) non-homologous end joining (NHEJ) pathway; (b) base excision repair (BER) pathway; (c) mismatch repair (MMR) pathway; (d) homologous recombination (HR) pathway; and (e) nucleotide excision repair (NER) pathway. These pathways are known to be active throughout the cell cycle, facilitating DNA damage repair (Chatterjee and Walker, 2017).

There are several types of DNA lesions, with both endogenous and exogenous sources of DNA damage. Photolesions are associated with UV-induced damage to the DNA. The photochemical reactions result in the production of cyclobutene pyrimidine dimers and pyrimidine 6-4 pyrimidone photoproducts. UV-induced DNA damage, however, is heterogeneous and influenced by epigenetic marks and the state of chromatin (Carusillo and Mussolino, 2020; Timmins, 2023). Double-strand breaks in the DNA are another type of DNA lesion, requiring repair for the preservation of chromosomal integrity. The repair of double-strand breaks is mediated by the HR pathway that involves single-strand annealing, replication, and gene conversion (Mehta and Haber, 2014). Single-strand breaks in the DNA, the most prevalent of the DNA lesions, may hinder the replication and transcription machinery of the DNA (Lin et al., 2019). Defective repair of single-strand breaks in the DNA causes the development of neurodegenerative or neurodevelopmental pathologies (Caldecott, 2024). DNA base lesions are characterized by the chemical modifications of nucleotide bases, which can be associated with single-strand breaks and nucleotide sugar modifications. DNA base lesions include hydrolysis, oxidation, alkylation, and deamination (Bauer et al., 2015).

DNA damage is critical to most aspects of the aging phenotype, which is rendered the primary cause of aging. The process of DNA damage occurs continuously throughout normal aging, following exposure to exogenous and endogenous genotoxins. Mutations and chromosomal aberrations, preceded by DNA damage, contribute to genomic instability (Schumacher et al., 2021). Genomic instability and associated induction of signaling cascades result in cellular senescence. Moreover, the DNA damage repair capacity declines with normal aging. Unrepaired DNA damage further reduces this potential, inhibiting the recovery of cells (Maynard et al., 2015). DNA damage and subsequent somatic mutations result in age-related cell function loss and disease associated with transcriptional noise (Vijg, 2021).

Furthermore, DNA damage accumulation is a key driver of aging, contributing to inflammation and age-related diseases (Zhao et al., 2023). This process is closely linked to nutrient-sensing pathways like mTOR-S6K, which regulates cell growth and metabolism (Antikainen et al., 2017). The mTOR-S6K pathway can impair DNA damage response by phosphorylating RNF168, leading to genome instability (Xie et al., 2018). Additionally, activation of the insulin/IGF-1 and mTOR signaling pathways promotes cell growth while inhibiting autophagy, a process associated with longevity (Temesgen Mitiku et al., 2024; Mitiku et al., 2024). Dysregulation of these pathways can result in accelerated aging and neurodegenerative diseases (Mitiku et al., 2024). The accumulation of DNA damage also triggers inflammation through mechanisms such as the cGAS-STING axis and NF-κB activation (Zhao et al., 2023). Understanding these interconnected processes may lead to potential interventions against age-related inflammation and diseases (Zhao et al., 2023; Antikainen et al., 2017).

Moreover, AMP-activated protein kinase (AMPK), a key metabolic sensor, regulates energy homeostasis, stress resistance, and cellular housekeeping (Salminen and Kaarniranta, 2012). It inhibits mTORC1 and promotes autophagy under low-energy conditions (Inoki et al., 2012). With age, AMPK activity declines, leading to impaired metabolic regulation, increased oxidative stress, and reduced autophagic clearance (Salminen and Kaarniranta, 2012; Morgunova and Klebanov, 2019). This age-related decrease in AMPK responsiveness is particularly critical for postmitotic cells (Morgunova and Klebanov, 2019). The resulting metabolic imbalance contributes to increased oxidative stress and pro-inflammatory signaling, activating innate immunity and triggering low-grade inflammation (Salminen and Kaarniranta, 2012). AMPK also regulates mitochondrial function, cell senescence, and longevity-related pathways (Ruiz et al., 2016). Understanding AMPK’s role in aging processes may provide insights into age-related diseases and potential interventions to promote healthy aging (Morgunova and Klebanov, 2019; Ruiz et al., 2016).

DNA damage and defective DNA repair are also implicated in inflammation and premature aging syndromes. Damaged DNA drives the activation of interferon response and chronic autoinflammatory response pathways, contributing to the development of premature aging phenotypes. Another mechanism by which DNA triggers inflammation is via proteins involved in DNA damage response. Moreover, telomere defects and persistent DNA damage stimulate senescence, which then results in the development of inflammation via the p38 pathway (Zhao et al., 2023).

Several biomarkers such as genomic, transcriptomic and proteomic factors can be utilized to measure biological age of individuals. Among the genomic markers DNA methylation aging clocks, DNAm pattern of 353 CpG sites, DNA methylation GrimAge, 73 CpG sites, 10 CpG sites, increase in DNAmAge, apolipoprotein E gene (*APOE*), and Forkhead box O3 gene (*FOXO3*) estimate biological age through respective mechanisms (Wu et al., 2021). Along with Trancriptomics aging clock, expression of eleven genes *PARP3*, *MANEAL*, *KIAA0408, AMZ1*, *ISM1*, *DDB2*, *CRIP1*, *NEFL*, *CHN1*, *CAPN2, and PHLDA3* is positively associated while *four genes SLC4A10, MXRA8, PLEKHA7, and CD248* are negatively associated with aging. *miR-21, Telomere-lncRNA, Meg3 and CircRNAs* are also involved in biological aging. Proteomics aging clocks, VEGFA, L-selectin, cystatin-C, alpha-2-HS-glycoprotein, Insulin-like growth factor-binding protein 2, serum protease inhibitors, coagulation factor X, alpha-2-antiplasmin, serum Albumin, Galectin-3, C-X-C motif chemokine 9 and 10, Apolipoprotein L1, plasminogen, fibronectin, and Telomerase are some of the proteomic biomarkers for detecting biological age (Kliuchnikova et al., 2024). In metabolomics, vitamin E metabolism, CoA catabolism, tryptophan metabolism, lysine metabolism, tyrosine metabolism, diacylglycerols, mono-acylglycerides, Nicotinamide adenine dinucleotide (NAD+), and Metabolic profile are indicators of cellular age (Wu et al., 2021; Srivastava, 2019).

Various machine learning models such as random forest, generalized linear model (GLM), gradient boosting model (GBM), K nearest neighbors (KNN), Logistics Regression and support vector machine (SVM) have been used to assess disease progression related to telomere length (Ruan et al., 2023).

### 2.2 Misfolded proteins

Proteins are critical to life functions and any change in the structure of proteins is reflected in their functioning. Under physiologic conditions, the proteins tend to be stable. This stability is achieved by the characteristic folding of the polypeptide chain to attain the native configuration. Both the primary sequence of the protein and the environment of the cell determine protein folding. While protein folding occurs successfully the majority of the time, unsuccessful folding results in the formation of misfolded proteins, which are implicated in several diseases (Zakariya et al., 2022; Ajmal, 2023).

The deviation of conformation from the native protein fold in misfolded proteins may arise due to several reasons. These include somatic mutations in the genome, translation or transcription errors, defective folding and chaperone machinery, impaired post-translational protein modifications, structural modifications of proteins induced by environmental factors, and protein misfolding mediated by seeding and cross-seeding mechanisms. Misfolded proteins tend to undergo self-aggregation, with the most favorable conformation being the ß-sheet structural motif. Small assemblies of these proteins eventually give rise to soluble oligomers, which induce cellular apoptosis. Larger misfolded protein aggregates tend to exhibit higher resistance to biological clearance compared to small aggregates of misfolded proteins (Moreno-Gonzalez and Soto, 2011). Both intracellular and extracellular factors influence the development of misfolded proteins. These include genetic mutations, changes in the pH, concentration of reactive oxygen species, and temperature (Devi et al., 2021). Other physiological risk factors that influence protein folding and functionality include salt and pressure (Devi et al., 2022).

Both genetic and environmental risk factors contribute to the misfolded proteins and their aggregation. These include heavy metals, pesticides, air pollutants, and other small molecules. Natural or synthetic small molecules such as denaturants, inhibitors, and activators contribute to the dysfunction of protein folding machinery. However, some small molecules such as doxycyclin, squalamine, and tea polyphenols have been associated with the inhibition of pathological aggregation of misfolded proteins (Devi et al., 2022; Dominguez-Meijide et al., 2021). Exposure to heavy metals and metalloids interferes with the process of protein folding and promotes the aggregation of misfolded proteins, which is implicated in impaired protein homeostasis and cell survival. The heavy metals implicated in the process of misfolding of proteins include cadmium and trivalent arsenic (Tamás et al., 2014). Notably, particulate air pollutants may also trigger or exacerbate the accumulation of misfolded proteins. Air pollution-mediated disruption of proteostasis promotes both inflammation and misfolding in neurodegenerative disorders (Kikis, 2020).

The aggregation of misfolded proteins is the hallmark feature of age-related disorders that involve impaired proteostasis, with neurodegenerative diseases being the most common of these disorders. Misfolded proteins are associated with progressive cellular damage and impaired organ functioning, with these features overlapping with the natural process of aging (Cuanalo-Contreras et al., 2022). Aggregation of misfolded proteins, endoplasmic reticulum stress, and impaired proteostasis are characteristic features of neurodegenerative disorders. Moreover, the normal process of aging is linked to changes in the expression of endoplasmic reticulum chaperones and folding machinery, resulting in the accumulation of misfolded proteins (Uddin et al., 2021). Besides physiological aging, the triggering of the endoplasmic reticulum stress response, mediated by the accumulation of misfolded protein, is linked to premature aging (Vidak et al., 2023). Mitochondrial dysfunction is also associated with impairment of protein homeostasis machinery whereas impairment of proteasome results in the exacerbation of mitochondrial dysfunction (Ross et al., 2024).

### 2.3 Telomere shortening/attrition

Telomere refers to repeats of non-coding nitrogenous bases that are present at the end of eukaryotic DNA chromosomes. These structures remain highly conserved and relatively unchanged through evolutionary changes (Lee and Pellegrini, 2025). The tandem nucleotide repeats include double-stranded regions at the proximal end and single-stranded regions at the distal end that confer protection to the chromosomal ends, maintaining genomic integrity. The stability of telomeres is attributed to shelterin complex proteins. The functions of the shelterin complex include regulation of telomere length, protection against chromosomal damage, and repression of DNA damage response signals. Other proteins associated with telomeres include chromosomal transcription factors and nucleosomes (Srinivas et al., 2020). Telomere shortening is a direct consequence of DNA replication and oxidative stress, which is countered by the catalytic activity of telomerase, an enzyme responsible for maintaining the length of the telomere (Saretzki, 2018).

Telomere length, regarded as a complex hereditary trait, is found to be associated with the process of aging as well as age-related diseases (Srinivas et al., 2020). Most of the epidimiological studies have conducted research on telomere length by using blood especially leukocytes or saliva as a genetic source. A very few number of studies have compared leukocyte with other human tissues observing that telomere length is variable in different tissue types (Sabharwal et al., 2018). Whole blood samples are taken as a proxy for all human tissues and telomerase activity depends upon TERT and TERC expression. However, at the older age due to lack of progenitor cells which are responsible for telomerase expression the TL is not indicative of TERT and TERC (Demanelis et al., 2020).

Telomere length, regarded as a complex hereditary trait, is found to be associated with the process of aging as well as age-related diseases (Srinivas et al., 2020). Telomere shortening or attrition is an established hallmark of cellular senescence and aging. Notably, age-related pathologies and disease processes exhibit telomere attrition at an accelerated rate. Therefore, telomere length is among the best biomarkers of aging. The correlation of telomere length with the chronological age of individuals throughout life, the predictive potential for morbidity and mortality, and reaction to harmful or beneficial exposures render telomere length a powerful biomarker of aging (Vaiserman and Krasnienkov, 2020). Events underlying telomere shortening and subsequent aging and age-related diseases are accounted for by the persistent activation of DNA damage response at the telomeres, resulting in replicative and cellular senescence. Moreover, the formation and accumulation of telomere-associated DNA damage response foci are also associated with aging-associated processes such as mitochondrial dysfunction, disruption of autophagy, impaired proteostasis, epigenetic dysregulation, and alteration of nutrient sensing (Rossiello et al., 2022).

While subsequent DNA replication contributes to progressive telomere shortening, concomitant with the normal aging processes, certain non-physiological processes also contribute to telomere attrition. Cellular exposure to oxidizing agents such as reactive oxygen species causes oxidative damage to the telomeres, which are intrinsically susceptible to oxidative damage. The G-rich sequence of telomeric DNA and the presence of shelterin complex at telomeres increase the likelihood of oxidative damage by mediating the development of DNA lesions or single-strand breaks and failure to recruit DNA damage response proteins. In turn, oxidized telomeres hinder the enzymatic activity of telomerase, preventing further extension of telomeres (Lin and Epel, 2022).

The influence of a number of factors such as age of the donor, socio-economic status, genetic and epigenetic predisposition, body weight, exercise and smoking on telomere length has been reported (Shammas, 2011). Women were observed to have longer telomeres compared to men in a study population which may be owing to x chromosome or hormones such as estrogen levels (Lapham et al., 2015). Dries et al. have reported that early biological aging and telomere length in a newborn baby is associated with socioeconomic status of parents (Martens et al., 2020). Furthermore, socioeconomic status, childbirth weight and maternal age also pose significant impact on telomere length in women (Assari et al., 2024). Children born in families with lower socioeconomic status have been reported to have shorter telomere length (Martens et al., 2020). Social behaviors and ethnicity have also been reported to play vital impact to telomere length (Vyas et al., 2021).

Modifiable lifestyle factors implicated in telomere shortening include smoking, insomnia, and physical activity (Chen et al., 2024). In addition to smoking and insomnia, alcohol is also related to telomere shortening, with the association being strongest among Hispanic and Black females (Vyas et al., 2021). Nutritional factors including vitamins, minerals, and bioactive components such as polyphenols and omega-3 fatty acids exhibit the potential to directly or indirectly influence the telomere length (Herrmann et al., 2018).

Epidemiological studies have demonstrated the relationship between mortality and telomere length in leukocytes, which is further linked to cardiovascular diseases and Alzheimer’s disease. Reduced leukocyte telomere length has been associated with increased risk for the development of age-related disorders including type 2 diabetes mellitus, premature aging syndromes, decreased bone mineral density such as in osteoporosis, and neurodegenerative diseases (Herrmann et al., 2018).

### 2.4 Epigenetic alteration

Epigenetics refers to heritable modifications in the DNA that alter gene expression without affecting the DNA sequence. The chemical modification of nitrogenous bases in the DNA is responsible for the regulation of gene expression under epigenetic mechanisms, hence, these mechanisms may influence both physiological and pathological processes (Al Aboud et al., 2025; Li, 2021). The major epigenetic mechanisms include DNA methylation, chromatin or histone modifications, and loss of non-coding RNA. Epigenetic modifications exhibit both mitotic and meiotic inheritance patterns (Hamilton, 2011).

DNA methylation is an epigenetic mechanism that involves the suppression of gene expressions and is also responsible for the regulation of histone modification and loss of non-coding RNA epigenetic mechanisms. In most instances, DNA methylation occurs at the CpG sites in a heterogeneous manner throughout the genome, forming CpG islands. The CpG islands and adjacent areas on the genome are of high functional significance given their role in the effective alteration of gene expression. Enzymes responsible for DNA methylation are DNA methyltransferases, which catalyze the transfer of a methyl group from S-adenosyl methionine to a cytosine residue, forming 5-methylcytosine (Kiselev et al., 2021).

Post-translational modifications of histones do not alter the DNA sequence like other epigenetic mechanisms; however, this mechanism is associated with the modified availability of the nucleotide sequence to the transcriptional process. Various different types of histone modifications exist such as ubiquitination, methylation, acetylation, and phosphorylation. The histone modifications predominantly occur at the *N-*terminal tails of histones and different modifications have different outcomes on transcription and gene expression. For instance, acetylation of histones is associated with elevated gene expression whereas methylation of histones may repress or promote transcription based on the number of methyl groups and location of the amino acid residues (Alaskhar Alhamwe et al., 2018).

Non-coding RNAs are referred to as clusters of RNA that are involved in post-transcriptional regulation of gene expression and are not involved in encoding functional proteins. Non-coding RNAs are classified as housekeeping and regulatory non-coding RNAs (Wei et al., 2017). Non-coding RNAs, predominantly microRNAs, and long non-coding RNAs, play a substantial role in the regulation of epigenetic mechanisms (Pathania et al., 2022). Non-coding RNAs are implicated in several biological processes including cellular apoptosis and proliferation. Moreover, non-coding RNAs serve as good diagnostic and prognostic indicators of metabolic pathologies and other disorders. Long non-coding RNAs are also involved in the regulation of DNA methylation and histone modification (Wu et al., 2023).

Epigenetic deregulation and associated alteration in the epigenetic events are evident during the process of aging. Epigenetic alterations that have a direct effect on the process of aging include the aggregation of histone variants, histone and heterochromatin loss, aberrant modification of histones, deregulation of non-coding RNAs, and altered chromatin accessibility. Epigenetic mechanisms contribute to the process of inflammation, which is a common occurrence during aging. Epigenetic mechanisms such as histone acetylation and ubiquitination are responsible for the regulation of NF-κB, associated with cellular senescence and apoptosis. Age-related disorders such as osteoporosis exhibit epigenetic modifications in mesenchymal stem cells. Similarly, neurodegenerative diseases including Alzheimer’s disease and Parkinson’s disease are associated with loss of heterochromatin, aberrant histone modification, and altered DNA methylation (Saul and Kosinsky, 2021). Various environmental factors and lifestyle habits such as excessive smoking, imbalanced diet, depression, weather changes, and stress reinforce epigenetic changes in gene expression, resulting in early DNA damage and premature aging (Dhar et al., 2022).

### 2.5 Cell cycle arrest

The transition of cells across arrested states and proliferative states determines the development and function of cells and tissues. The divergence of cells from the proliferative state of the cell cycle results in their progression to quiescent and senescent cell cycle arrest states, which are reversible and irreversible, respectively (Stallaert et al., 2022). The senescent state represents stable cell cycle arrest, which occurs in response to development signals and other internal and external stimuli. This form of cell cycle arrest is unresponsive to mitogenic signals, however, senescent cells exhibit alteration in metabolic processes and may undergo changes in gene expression. The arrest of the cell cycle in senescent cells is predominantly mediated by the activation of either of the tumor suppressor pathways–p53/p21 pathway and p16/pRB pathway. Given that senescent cells do not proliferate but remain metabolically active, these cells exhibit a secretory phenotype, which enables their communication with the neighboring cells. The hypersecretory phenotype of senescent cells is referred to as the Senescence Associated Secretory Phenotype (SASP). The SASP components include soluble signaling factors and extracellular matrix components (Kumari and Jat, 2021).

The cell cycle progression is mediated by cyclin-dependent kinases (CDKs), which are serine/threonine protein kinases responsible for the phosphorylation of substrates, mediating DNA synthesis and progression of mitosis. While binding to cyclins allows the activation of CDKs, the activity of these kinases can be inhibited by tyrosine phosphorylation of binding of small inhibitory proteins. Cell cycle checkpoints are critical to the surveillance of the cell cycle, which is implicated in cell size control, DNA damage responses, monitoring of DNA replication, and mitotic spindle checkpoint (Barnum and O’Connell, 2014). Given that damaged DNA can propagate through the S-phase and mitotic phases of the cell cycle, cell cycle arrest may occur at the G1 and G2 phases to prevent the replication of damaged DNA and the segregation of aberrant chromosomes, respectively. The spindle checkpoint, present in the mitotic phase of the cell cycle, ensures equal segregation of the chromosomes. Mitotic spindle damage or the lack of attachment of chromosomes to microtubules results in spindle checkpoint-mediated inhibition of the cell cycle progression (Blagosklonny and Pardee, 2002).

Senescent cells exhibit an increase in the expression of CDK inhibitors, which are also known as cell cycle-inhibitory proteins. The most prominent of the CDK inhibitors associated with the accumulation of senescent cells as part of the aging phenomenon is p16^INK4A^. The upregulation of cell cycle inhibitors, p16 and p21, oxidative damage and the associated increase in the levels of reactive oxygen species, upregulation of anti-apoptotic proteins, SASP, and prolonged activation of DNA damage response are all characteristic features of senescent cells (Di Micco et al., 2021). The cell cycle arrest of senescent cells is known to occur in the G1 phase of the cell cycle, inhibiting the initiation of the DNA replication process in these cells. While senescent cells are thought to exist in irreversible G1 phase arrest, the mechanism of senescence may also initiate during the G2 phase of the cell cycle in response to damage to telomeres, DNA damage, or activation of oncogenes. Therefore, senescent cells exit the cell cycle at the G1 and G2 phases, which is further associated with the activation of p53 and RB/P16 pathways (Roger et al., 2021).

### 2.6 Stem cell exhaustion

Stem cells play an important role in tissue homeostasis, which is maintained and regulated within a microenvironment or niche, forming a regenerative unit. The regenerative units of stem cells respond to injury and other external stimuli. Adult stem cells, which mostly exist in a quiescent state, are derived from their embryonic counterparts, which are known to undergo active proliferation (Brunet et al., 2023). The adult tissue stem cells are critical to the maintenance of tissue homeostasis and repair. These stem cells may undergo asymmetrical cell division, giving rise to both stem cells for replacement purposes or self-renewal and cells with specialized functions. Since adult stem cells exist in a quiescent state and hold the potential for proliferation, the manipulation of these stem cells can facilitate the treatment of age-related degenerative disorders (Rando and Jones, 2021). The imbalance between quiescence and proliferation of stem cells culminates into stem cell exhaustion, which is accelerated by failure to regulate the cell cycle (Oh et al., 2014).

Stem cell exhaustion, a hallmark of aging, is driven by several mechanisms. Telomere shortening is critical to the age-related exhaustion of stem cells. The gradual decrease in telomere length with each replication cycle results in the senescence of cells, limiting the replicative potential of stem cells. Notably, telomere length can be utilized as an indicator of the proliferative potential of stem cells. Moreover, significant telomere attrition results in the activation of DNA damage response and apoptotic signaling pathways, resulting in cell cycle arrest and senescence in the stem cells (Fathi et al., 2019). The age-related accumulation of reactive oxygen species also drives stem cell dysfunction. Reactive oxygen species accumulation and associated impairment in cellular antioxidant mechanisms also contribute to stem cell dysfunction (Oh et al., 2014). Epigenetic alterations are another mechanism driving age-related stem cell dysfunction. These alterations include aberrant histone modifications. Age-related changes in histone methylation are associated with age-related changes in gene expression. Pan-acetylation of histone proteins is also associated with age-related senescence of mesenchymal stem cells (Sun et al., 2023).

The age-related decline in the stem cells is also associated with mitochondrial dysfunction. The accumulation of mutations in mitochondrial DNA in human-induced pluripotent stem cells is found to be higher in cells derived from elderly individuals compared to their younger counterparts. Mitophagy or selective degradation of mitochondria that are dysfunctional is impaired during aging (Wan and Finkel, 2020). Furthermore, DNA damage has several cumbersome effects on stem cells. The accumulation of mutations, induction of apoptosis and senescence, and shortening of telomere length contribute to the senescence of stem cells. The depletion of stem cells is closely linked to the phenomenon of aging and the development of age-related diseases (Mi et al., 2022). Adult stem cell exhaustion is further associated with the onset of premature aging, characterized by graying of hair, reduction in fat mass, and increased kyphosis (Vilas et al., 2018).

### 2.7 Intercellular communication

Intercellular communication is a characteristic feature of both physiological and pathological processes. Intercellular communication refers to the mechanisms employed by the cells to communicate with other cells, which is implicated in the maintenance of tissue homeostasis. The intercellular communication across senescent cells, mediated by SASP, is a complex process (Fafián-Labora and O’Loghlen, 2020). Dysregulated intercellular communication is considered a hallmark feature of aging, associated with the age-related emergence of cell-to-cell stochasticity. The intercellular deregulations may include impairment of immune surveillance, extracellular matrix remodeling, age-related chronic inflammation, increase in the levels of SASPs, and modification in the communication across stem cells and the niche (Lagger et al., 2023).

The different types of intercellular communication include direct cell-cell contact, paracrine signaling, endocrine signaling, and autocrine signaling. Contact-dependent intercellular communication refers to the direct contact between cellular membranes. Gap junctions are described as specialized cell-cell junctions, which allow the intercellular exchange of small signaling molecules without any intervening plasma membranes. Paracrine signaling depends on the release of signaling molecules into the extracellular space, which acts on adjacent cells. On the contrary, endocrine signaling involves the release of hormones into the blood, with target cells distributed throughout the body. Lastly, autocrine signaling involves the release of signaling molecules, which bind to receptors of the same cell. Autocrine signaling is reinforced during the process of development whereby cells employ autocrine signaling to undergo a specific differentiation pathway (Alberts, 2002).

There are several changes in intercellular communication associated with the process of aging. Aging-mediated pathological gap junction remodeling is linked to altered gene expression and post-translational connexin modifications (Zeitz and Smyth, 2023). Growth factors, cytokines, injury, blood glucose levels, and other physical and chemical factors affect the pattern of connexin expression in the gap junctions (Gong et al., 2021; Yeh et al., 2000). The normal phenomenon of aging also involves the alteration of endocrine signaling pathways, which are also further associated with the regulation of the aging process. This may be linked to caloric restriction, which mediates modifications in the levels of endocrine signaling (Allard and Duan, 2011). The two characteristic age-related changes in hormonal signaling include altered secretion of hormones by the hypothalamic-pituitary-adrenal axis and a decline in the sensitivity of this central hormonal axis to hormones and feedback loop pathways. In addition to this, the target tissues may also exhibit a decline in their sensitivity to hormones with age (Hill et al., 2020). Similarly, age-associated changes in the brain that contribute to cognitive decline may emerge as a result of altered neurotransmitter signaling and the accumulation of damaged tissue. The microscopic changes occurring in the brain as part of the normal aging process include the accumulation of lipofuscins, which affect the synthesis of neurotransmitters in the brain (Lee and Kim, 2022).

Disruption of intercellular communication across parenchymal, stromal, and immune cells plays a critical role in the development of diseases and pathological processes (Berumen Sánchez et al., 2021). Adequate and appropriate intercellular communication across immune cells plays a pivotal role in the generation of coordinated immune responses, which can be mediated by extracellular vesicles (Pelissier Vatter et al., 2021). Tissue injury and stress states culminate in the alteration of intercellular communication mediated by the extracellular vesicles, resulting in the emergence of disease states (Berumen Sánchez et al., 2021).

### 2.8 Nutrient-sensing pathway

The nutrient-sensing pathways are critical to the process of aging, given the role of nutrients in the direct and indirect activation of different cellular pathways. Several genes implicated in lifespan regulation are also involved in nutrient-sensing pathways. These genes are known as nutrient-sensing longevity genes. The role of nutrient signaling pathways in aging and lifespan regulation can be explained by the importance of nutrients in the maintenance of energy homeostasis. The nutrient-sensing pathways are biochemical pathways that sense the availability of nutrients (Davinelli et al., 2012). Nutrient-sensing pathways mediate anabolism and storage of nutrients in cases of food abundance whereas autophagy, mobilization of internal nutrient stores, and other homeostatic mechanisms are activated in cases of nutrient scarcity. The nutrient-sensing mechanisms are deregulated in several diseases including metabolic disorders (Efeyan et al., 2015).

Classical nutrient-sensing pathways include the insulin-like growth factor 1 (IGF-1) signaling pathway, the AMP-activated protein kinase (AMPK) pathway, and the mammalian target of rapamycin (mTOR) pathway. Nutrient signaling mediated by IGF-1/insulin can be deregulated during the process of aging, mediating the onset of chronic inflammation. Loss-of-function mutations in the somatotropic axis components including IGF-1 and growth hormone tend to affect longevity. Proper IGF-1 signaling is significant for supporting immune homeostasis, particularly in the older population. The deregulation of this nutrient-sensing pathway is linked to immunosenescence (Bettedi and Foukas, 2017; Baechle et al., 2023). The mTOR nutrient-sensing pathway orchestrates the balance between anabolic and catabolic mechanisms across various metabolic states. Specific stimuli associated with the activation of the mTOR pathway include mechanical stimuli and amino acid deprivation (Liu and Sabatini, 2020). Suppression of the IGF-1 signaling pathway/mTOR pathway drives the beneficiary outcomes of dietary restriction, prolonging the lifespan and delaying the onset of age-related diseases. The age-related processes linked to aberrant mTOR signaling include loss of proteostasis, mitochondrial dysfunction, cellular senescence, and stem cell dysfunction (Papadopoli et al., 2019). The AMPK nutrient-sensing pathway is an important cellular energy sensor, which is triggered by low energy levels. The AMPK pathway responds to the stimulus by promoting ATP production and catabolic protein expression. Moreover, this nutrient-sensing pathway is also involved in the conservation of energy via the regulation of non-metabolic mechanisms and switching off anabolic pathways (Hardie et al., 2012). The AMPK pathway also regulates intracellular signaling pathways that control cell growth, cellular senescence, autophagy, and cell growth. The age-related decrease in the activation and responsiveness of the AMPK pathway increases susceptibility to cellular stress and reduces autophagic clearance, therefore, strategies increasing AMPK activation are implicated in lifespan prolongation (Stancu, 2015).

Several lifestyle factors have a significant effect on aging, which is mediated by aberrant nutrient-sensing pathways. Lifestyle factors that activate or suppress nutrient-related signaling pathways include diet, smoking status, lack of physical activity, and a sedentary lifestyle. Other modifiable environmental risk factors include reduced sleep, psychosocial stress, and high fat content in the diet, accelerating aging and promoting the development of age-related diseases. The dysregulation of the nutrient-sensing network is an established pathological mechanism underlying metabolic diseases. A type of non-coding RNA, microRNAs, target genes that encode proteins and enzymes linked to the nutrient-sensing pathways, contributing to the process of aging. MicroRNAs that modulate the IGF-1 and mTOR pathways may serve as pharmacological targets for delaying the process of aging (Micó et al., 2017).

## 3 Role of telomere abnormalities in metabolic diseases

**FIGURE 1:**
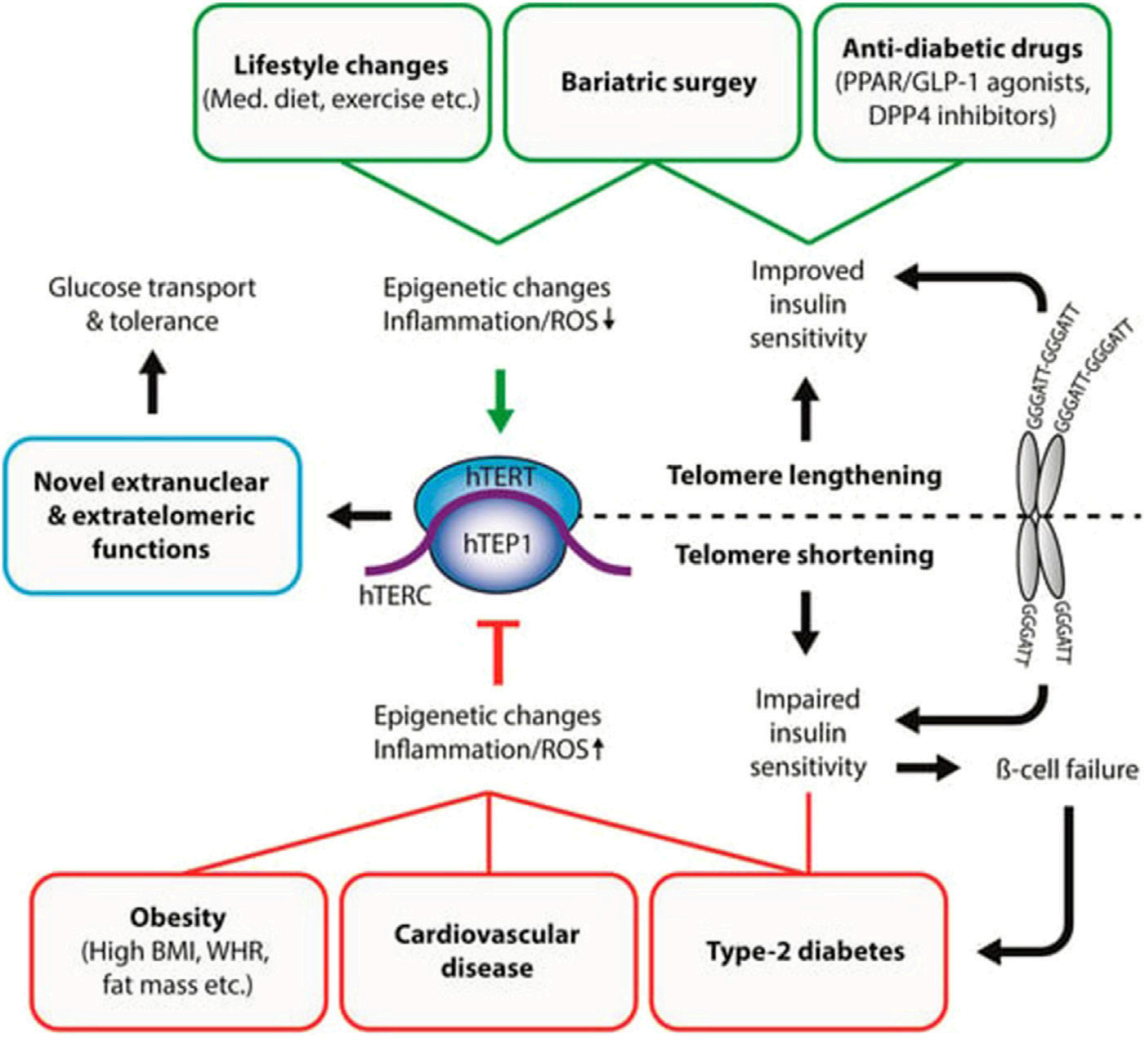
Interaction between disease states and telomerase activity. The relationship between telomere abnormalities and metabolic diseases is complex and multifaceted (Figure 1). This figure shows that telomere lengthening, healthy lifestyle (diet, exercise) modifications, anti-diabetic drug prevent or delay telomere shortening and reduce the risk of metabolic disorders (Type-2 diabetes, obesity, cardiovascular diseases), and developing therapeutic interventions that target telomere maintenance. BMI: body mass index; WHR: waist-to-hip ratio; Med. Diet: Mediterranean Diet; ROS: reactive oxygen species; hTERT: human telomerase reverse transcriptase; hTERC: non-coding telomerase RNA component; hTEP1: human telomerase-associated protein 1; PPAR: peroxisome proliferator-activated receptor; GLP-1: glucagon-like peptide 1; DPP4: Dipeptidyl peptidase-4. Source: Kirchner et al. (2017). Permission granted by MDPI.

The relationship between telomere abnormalities and metabolic diseases is complex and multifaceted (Figure 1). A thorough understanding of this relationship has important implications for early detection of at-risk individuals for metabolic diseases based on telomere length, promoting healthy lifestyle modifications to prevent or delay telomere shortening and reduce the risk of metabolic disorders, and developing therapeutic interventions that target telomere maintenance.

As a strong indicator of biological age detection, telomeric length is often associated with age-related and metabolic diseases. Telomeric length is sensitive to early life instances, such as maternal hormonal, metabolic, inflammatory, or oxidative stress exposures which program telomere biology leading to increased biological aging. Several epidemiological studies have shown telomere length to be associated with age-related diseases (Daios et al., 2022). For instance, telomere length is shorter promoting atherosclerosis in coronary heart disease (Herrmann and Herrmann, 2020). Higher leukocyte telomere length shortening rate is also associated with hypertension (Liu et al., 2019), type 2 diabetes mellitus (Krasnienkov et al., 2018), metabolic syndrome (Khalangot et al., 2020), and osteoarthritis (Fragkiadaki et al., 2020). Consequently, it is reported that telomere length can be utilized as a biomarker for diseases such as endometrial cancer (Benati et al., 2020), cardiovascular (Sagris et al., 2022) and Alzheimer’s disease (AD) (Corbo et al., 2021). Alzheimer’s driven Brain changes are reported by many studied to be corelated to shorter leucocyte telomere length (LTL) associated with memory and cognitive functioning (Topiwala et al., 2023). However, contradictory results have been shown in other studies where LTL was uninfluenced by AD which raises a question if LTL can be used as a diagnostic biomarker (Hinterberger et al., 2017).

### 3.1 Type 2 diabetes mellitus

**FIGURE 2:**
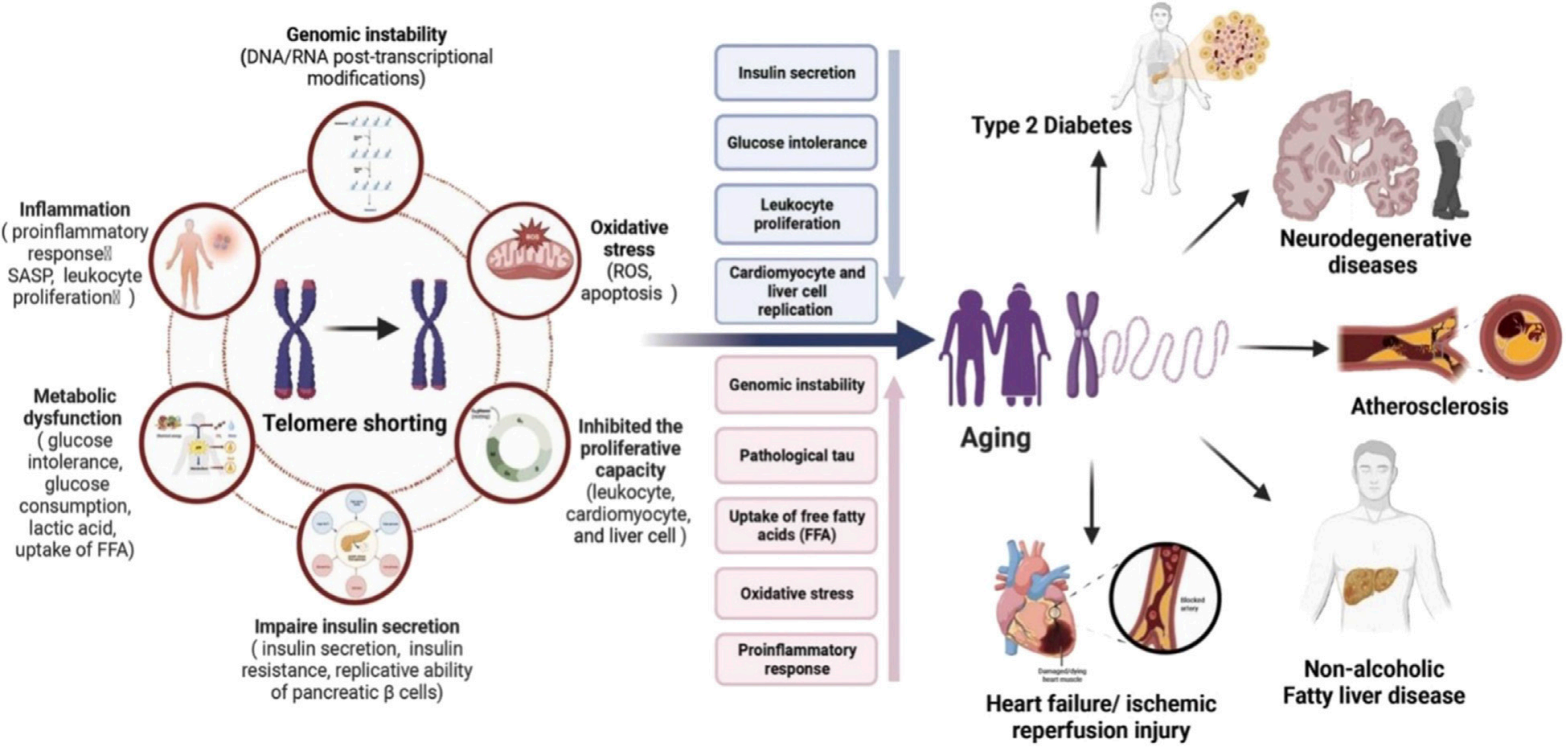
Activities of telomerase play distinct roles in the development of age-related chronic diseases. Telomerase shortening is linked with various conditions like genomic instability, oxidative stress, inhibit proliferative capacity, impair insulin secretion, metabolic dysfunction, and inflammation and these condition ultimately responsible for increasing aging process, Type-2 diabetes, neurodegenerative diseases, atherosclerosis, non-alcoholic fatty liver diseases, heart failure, ischemic reperfusion injury, etc. Source: “Reproduced from Wang et al. (2023). Elsevier Masson SAS. All rights reserved.”

Type 2 diabetes mellitus (T2DM), a chronic metabolic disorder characterized by persistently elevated blood glucose levels, is found to have a complex relationship with telomere biology. It has been demonstrated that individuals with T2DM have leukocyte telomere shortening compared to individuals without T2DM. Studies have also hypothesized that the worsening of diabetes is associated with the shortening of telomeres and subsequent cellular senescence and apoptosis (Chaithanya et al., 2023). Shortening of telomeres, a hallmark of aging, is associated with both diabetes and diabetes-related complications (Cheng et al., 2021) (Figure 2). The telomeric length may, however, differ across the types of diabetes including T2DM, latent autoimmune diabetes in the young (LADY), and latent autoimmune diabetes of adulthood (LADA). T2DM is found to have relatively longer telomeres compared to patients with LADA, which is attributed to the protection conferred by insulin and metformin (Cuevas Diaz et al., 2023). Insulin resistance and increased oxidative stress in individuals with T2DM are linked to the shortening of telomeric length, with the association being further strengthened by the link between improved glucose control, reduced insulin resistance, and prolongation of telomeres (Cuevas Diaz et al., 2023; Ma et al., 2015). Notably, DNA damage associated with critical telomere attrition is known for direct triggering of adipocyte insulin resistance (Loh et al., 2021). Leukocyte telomeric length is found to have a negative correlation with glucose tolerance, predominantly fasting plasma glucose levels in T2DM (Khalangot et al., 2020).

Chronic hyperglycemia and elevated HbA1c levels are negatively linked to the length of telomeres, indicating the potential association between higher glucose levels and telomere attrition. Moreover, the accumulation of advanced glycation end products (AGEs) results in the production of pro-inflammatory metabolites, driving telomere shortening. Shorter leukocyte telomere length in the setting of insulin resistance is linked to low adiponectin levels, increased adipocyte mass, and higher levels of C-peptide (Chaithanya et al., 2023). A case-control candidate gene association study conducted by Sethi *et al.* evaluated various telomere maintenance gene variants, demonstrating a significant association between T2DM and five gene variants including the *OBFC1, TERF2, TERC/LRRC31, TERC/MYNN*, and *CSNK2A2*, with no significant correlation with the age of participants (Sethi et al., 2020). A prospective Mendelian randomization analysis by Cheng *et al.* demonstrated a statistically significant association between an elevated risk of glycemic progression and shorter relative leukocyte telomeric length in T2DM patients. Short telomeres may trigger both *in vivo* and *in vitro* defects in spontaneous insulin secretion, resulting in impaired cellular proliferation and activated DNA damage response (Cheng et al., 2022).

The association between gene polymorphisms of telomerase reverse transcriptase (TERT) variant rs2736100 and the risk of T2DM in the Iraqi population was investigated in a case-control study by Alwan *et al.* The study findings indicated that the frequency of homozygous genotypes was higher in T2DM patients compared to control group participants, however, the heterozygous genotype was less frequent in T2DM patients. Moreover, the homozygous genotypes had an increased association with the risk of T2DM whereas the opposite was observed for the association between heterozygous genotype and the development of T2DM. The rs2736100 variant of the *TERT* gene was previously found to be associated with the length of telomere and disorders including T2DM (Alwan and Research, 2023).

### 3.2 Obesity

As highlighted previously, leukocyte telomere length is associated with aging and age-related diseases including obesity. The telomeric shortening is accentuated by oxidative stress and inflammation, which is negatively correlated with obesity. Both obesity and truncal subcutaneous adiposity are demonstrated to be the major factors underlying telomere shortening in individuals with impaired fasting glucose levels (Bhatt et al., 2022). Moreover, overweight or obesity are also known to progressively drive the shortening of telomeric length in women with polycystic ovary syndrome (PCOS). The association between obesity and telomeric length is further demonstrated by the increase in leukocytic telomere length in normal-weight women with PCOS (Kogure et al., 2024). The National Health and Nutrition Examination Survey (NHANES) analysis has demonstrated the synergistic effect of sarcopenia and obesity on telomere attrition, with significantly shorter telomeres linked to high adiposity-low muscle mass compared to low adiposity-high muscle mass (Goddard et al., 2022).

The association between obesity and leukocyte telomere length is driven by several mechanisms including oxidative stress, chronic inflammation, and metabolic dysfunction. Oxidative stress is considered a common pathway connecting obesity with relevant complications. G protein-coupled receptor kinase 2 is implicated in endothelial dysfunction, linking obesity with oxidative stress. Oxidative stress also drives dysregulation and suppression of autophagy, resulting in the development of an obesogenic environment. Even in younger individuals, telomere length is decreased in association with obesity and stress with total antioxidant capacity and total oxidation status significantly related to obesity in these patients (Martínez-Martínez and Cachofeiro, 2022). Moreover, relative telomere length is also associated with metabolically unhealthy obesity. Patients with metabolic dysregulation may exhibit shortening of telomeres, which is linked to oxidative stress and obesity in metabolic syndrome (Lejawa et al., 2021). Another mechanism by which central obesity decreases telomere length is via inflammatory markers such as C-reactive proteins and adipokines. Notably, a diet high in fat and low in fiber content, indicative of high inflammatory potential, is associated with shorter leukocyte telomere length (Zadeh et al., 2023).

Telomere maintenance genes including *TERC* variant rs12696304G/C minor allele frequency are found to be significantly low in patients with a normal body mass index (Giha et al., 2022). In cases of pediatric obesity, a significant increase is observed in DNA methylation in 13 CpG sites in obese children compared to children with normal weight. The expression of TERT is mediated by cytokines related to NF-κB and PI3K/AKT signaling pathways. Epigenetic alterations including hypermethylation of DNA at the *TERT* promoter site repress the transcription of promoter alleles (Liu et al., 2021). Telomerase RNA template, *TERC* gene is widely expressed in somatic cells, which is associated with the silencing of telomerase enzyme. Cytosolic *TERC-53* is responsible for the transmission of signals of mitochondrial dysfunction to the cellular nucleus. The binding of nuclear *TERC* directly to promoter sites is further associated with an increase in the transcription of genes implicated in the development of inflammatory responses (Moustakli et al., 2024). The *POT1* gene encodes the protection of telomeres protein 1, which is responsible for the protection and regulation of telomeric DNA. The expression of the gene and gene paralogs *POT1a* and *POT1b* can be enhanced with lifestyle interventions including exercise. The expression of the *POT1b* gene is found to be significantly higher in young mice given a low-fat diet compared to young mice given a high-fat diet (Bloom et al., 2020).

### 3.3 Neurological disorders

The role of telomere attrition has been investigated in several neurological disorders including neurodegenerative disorders and disorders with a predominant genetic basis. It is, however, observed that the rate of telomere shortening is relatively shorter in brain tissues compared to other body tissues, which may be attributed to telomere maintenance mechanisms in brain stem cells (Anitha et al., 2019). The major focus in neurodegenerative disorders, in the context of telomere shortening, has been Alzheimer’s disease and Parkinson’s disease. Amyloid-ß deposition in Alzheimer’s disease causes microglia activation and dystrophy, however, telomere shortening has been demonstrated to mitigate the progression of amyloid plaque pathology by influencing microglial activation. Moreover, the absence of *TERT* is related to higher levels of phosphorylated tau, resulting in increased damage to the neurons by reactive oxygen species. Studies have also demonstrated the correlation between telomere attrition and cognitive decline as well as progression to Alzheimer’s disease from mild cognitive impairment (Levstek et al., 2020). The role of telomere length in disease onset and progression is, however, not established in Parkinson’s disease as both short and long telomeres have been demonstrated in these patients. Moreover, there are contradictory findings related to the correlation between telomere length and gene expression of *CDKN2A* in Parkinson’s disease patients (Asghar et al., 2022).

Telomere attrition has been emerging as a potential contributor to the development of various other neurological disorders including schizophrenia and autism spectrum disorders (Anitha et al., 2019) (Figure 2). Shorter telomere length in children with autism spectrum disorders compared to typically developing children is associated with biological mechanisms underlying sensory symptoms of these disorders, with the degree of telomere attrition being associated with the severity of sensory symptoms. Furthermore, telomere length is related to cognitive function in parents of autistic children, but not in children and adolescents (Lewis et al., 2020). The high heritability of telomeres increases the risk for age-related pathologies in children with shorter telomeres at the time of birth. The telomere length also contributes to various childhood neurodevelopmental disorders including attention-deficit/hyperactivity disorder (ADHD). Children and maternal telomere length, but not paternal telomere length, have been demonstrated to have an association with the predominant hyperactive-impulsive ADHD type. Moreover, telomere length is a potential biomarker of the symptomatic burden of ADHD (Costa Dde et al., 2015).

Progeria refers to the clinical manifestations of premature aging, and progeroid syndromes constitute a group of heterogeneous genetic disorders with a phenotype of premature physiological aging. Progeroid syndromes may include ataxia-telangiectasia, xeroderma pigmentosum, Werner syndrome or adult progeria, and Hutchinson-Gilford syndrome (Sinha et al., 2014). Hutchinson-Gilford syndrome, the most studied progeroid syndrome, arises due to a mutation in the lamin A gene or *LMNA*, resulting in the production of mutant LMNA protein or progerin. Fibroblasts from Hutchinson-Gilford syndrome patients demonstrated aberrant histone modification, altered gene expression, and delayed DNA damage repair response. Progerin production and damage to the telomeres are known to act synergistically to induce cellular aging in Hutchinson-Gilford syndrome (Sinha et al., 2014; Cao et al., 2011). The expression of the mutant gene *LMNA* is required for loss of telomeric length in this syndrome and it has been demonstrated that telomere length remains within the normal range in cells that do not express lamin A protein in Hutchinson-Gilford syndrome patients (Decker et al., 2009). Telomere dysfunction mediated by progerin protein leads to transcription of telomeric non-coding RNAs, activating DNA damage response and premature cellular senescence in cellular systems in Hutchinson-Gilford syndrome (Aguado et al., 2019).

### 3.4 Liver cirrhosis

Liver cirrhosis, sequelae of chronic hepatic damage and inflammation, can be described as diffuse hepatic fibrosis and regenerative liver nodules. Regarded as the end stage of chronic liver disease, hepatic cirrhosis may arise from several causes including alcohol intake, hepatitis viruses, and autoimmune conditions (Liu and Chen, 2022; Tapper and Parikh, 2023). One of the pathophysiological mechanisms underlying the development of liver diseases including cirrhosis is oxidative stress, which is associated with structural and functional impairment in the liver. The generation of reactive oxygen species and oxidative stress in viral hepatitis and alcohol liver diseases contribute to the hepatic fibrogenic response, yielding progressive liver damage (Cichoż-Lach and Michalak, 2014). Oxidative stress is tightly correlated with inflammation, contributing to the development of a vicious cycle, leading to liver cirrhosis and hepatocellular carcinoma. Moreover, excessive inflammatory cells release reactive oxygen species and reactive nitrogen species, which further promote the expression of genes encoding pro-inflammatory cytokines (Pomacu et al., 2021). Aging is associated with an increase in the incidence of chronic liver diseases, and worsening of disease prognosis, and is considered a risk factor for the development of chronic liver diseases at different stages (Figure 2). The process of aging is implicated in the reduction of hepatic mass and blood flow, affecting its functionality. Healthy aging is also associated with a moderate risk of hepatic vascular resistance, reducing hepatic perfusion (Maeso-Díaz and Gracia-Sancho, 2020).

Senescence is a potential mechanism underlying the pathogenesis of chronic liver diseases. Senescent cells promote the accumulation of fat in the liver as well as steatosis. Fat accumulation is associated with the shortening of telomeres and DNA damage, which may be driven by oxidative stress. In the later stages of chronic liver disease, the senescent hepatocytes secrete excessive SASP factors, which contribute to telomere attrition in the hepatocytes (Maeso-Díaz and Gracia-Sancho, 2020). Notably, progressive hepatic fibrosis in chronic liver disease due to chronic inflammation results in replicative hepatocyte senescence. This mechanism is responsible for linking the length and maintenance of telomeres with the development of cirrhosis. Shortened leukocyte telomere length is associated with both advanced liver disease in the elderly population and a significantly greater risk of mortality in patients with liver diseases. The frequent replication of hepatocytes in diseased states is hypothesized to increase the shortening of telomeres in hepatocytes. Moreover, advanced liver disease is associated with higher rates of telomere attrition (Rattan et al., 2022). Telomere shortening is also referred to as a genetic factor underlying the risk of cirrhosis development. Moreover, telomerase *TERT* and *TERC* mutations are demonstrated to be associated with various etiologies of cirrhosis including viral hepatitis and alcohol-induced cirrhosis. The restoration of telomerase via the telomerase RNA gene is implicated in the reduction of fibrosis and improvement in hepatic functioning (Chaiteerakij and Roberts, 2011).

### 3.5 Chronic kidney disease

Chronic kidney disease can be described as persistent abnormality in renal structure and function for greater than 3 months, characterized by glomerular filtration rate <60 mL/min/1.73 m^2^ or albuminuria greater than or equal to 30 mg per 24 h. In developed nations, chronic kidney disease is associated with hypertension and diabetes. The genetic factors that contribute to the risk of chronic kidney disease include sickle cell trait and *APOL1* alleles (Chen et al., 2019). Older age is one of the predictors of chronic kidney disease, with a substantial proportion of the elderly population suffering from chronic kidney disease. It is rather a matter of debate whether or not age-associated decline in the glomerular filtration rate is associated with the normal aging process. Nonetheless, the preservation of renal hormonal function, acid-base balance, and electrolyte balance in older adults with glomerular filtration rates <60 mL/min/1.73 m^2^ suggests that this is part of physiological aging (Mallappallil et al., 2014).

Chronic kidney disease is increasingly recognized to be linked to accelerated aging among other characteristic features such as decline in cognition, quality of life, physical performance, and appetite (Merchant and Vathsala, 2022). The common features of chronic kidney diseases and aging are oxidative stress and inflammation. Oxidative stress and resultant production in mitochondrial reactive oxygen species, along with the inactivation of antioxidant enzymes results in the release of cytochrome c from mitochondria into cytosol, inducing cellular inflammation and apoptosis. This inflammatory response is also related to the activation of NLRP3 inflammasome and cGAS-STING pathway following leakage of mitochondrial DNA. The cGAS-STING pathway functions as a cytosolic DNA sensor, identifying the presence of double-stranded DNA in the cytoplasm and subsequently activating the NLRP3 inflammasome, which in turn causes the production of pro-inflammatory cytokines like interleukin-1 beta (IL-1β). The NLRP3 inflammasome is a crucial protein complex within the innate immune system that causes inflammation in response to various cellular stressors, including pathogen-associated molecular patterns (PAMPs) and damage-associated molecular patterns (DAMPs). Several different molecular and cellular mechanisms are implicated in kidney aging and chronic kidney disease. DNA methylation within CpG dinucleotides is involved in the progression of chronic kidney disease, the reversal of which may alleviate disease progression. Histone acetylation and methylation have also been implicated in kidney aging and chronic kidney disease. Another epigenetic mechanism implicated in chronic kidney disease is the upregulation of microRNAs in the renal cortex. The non-coding RNAs promote inflammation, apoptosis, and senescence via the TGF-ß/Smad signaling pathway (Zhang et al., 2024).

Increased telomere attrition in chronic kidney disease and renal fibrosis is attributed to several mechanisms including immune deficiency, oxidative stress, and inflammation. Reduced telomere length is associated with a reduction in the tissue repair potential of kidneys in response to injury, increasing susceptibility to chronic kidney disease or exacerbating existing disease. Telomerase-deficient animal models have been demonstrated to develop renal fibrosis and severe renal dysfunction. Moreover, telomere attrition or shelterin dysfunction predisposes the deprotected telomeres to epigenetic alterations including non-coding RNAs (Akinnibosun et al., 2022). Kidney replacement therapies such as hemodialysis and renal transplantation have been investigated for their association with telomere length. Increased inflammation and iron overload in hemodialysis with associated oxidative stress leads to accelerated telomere length shortening in patients undergoing hemodialysis (Levstek and Trebušak Podkrajšek, 2023).

## 4 Therapies for telomere disease

Telomere diseases or human telomere syndromes, characterized by the shortening of telomeres, are common premature aging disorders. The well-known telomere disorders are short telomere syndromes, with a wide spectrum based on the variation of defects. The emergence of short telomere syndromes in adulthood is likely to be associated with an age-related disorder, idiopathic pulmonary fibrosis. In up to half of the cases of short telomere syndromes, the culprit is the autosomal dominant-inherited heterozygous mutations in the *TERT* gene, which exhibits the phenomenon of anticipation, driven by the short telomere length. Short telomere premature aging phenotypes are associated with the development of disease in high-turnover tissues among children and young adults whereas disease development involves low-turnover tissues in adults (Armanios, 2022). Mutations in genes responsible for telomere maintenance *give* rise to several telomere syndromes including dyskeratosis congenita, liver fibrosis, and Hoyeraal-Hreidarsson syndrome. Telomere attrition of stem cells is considered one of the primary molecular mechanisms underlying aging and the development of age-related disorders (Martínez and Blasco, 2017). Premature telomere attrition, as a consequence of germline mutations in the genes encoding for proteins involved in the repair and maintenance of telomeres, mediate the development of both telomeropathies and telomere syndromes (Martínez and Blasco, 2017; Stanley and Armanios, 2015). Various previous studies showed the effectiveness of telomerase-targeted therapy in preclinical and clinical models (Tao et al., 2024). Targeting Telomere Dynamics as an Effective Approach for the Development of Cancer Therapeutics (Waksal et al., 2023), Telomerase-targeted therapies in myeloid malignancies (Ali and Walter, 2023), Combining old and new concepts in targeting telomerase for cancer therapy: transient, immediate, complete and combinatory attack (TICCA) (Wang et al., 2024), Clinical research progress of telomerase targeted cancer immunotherapy: a literature review).

**FIGURE 3:**
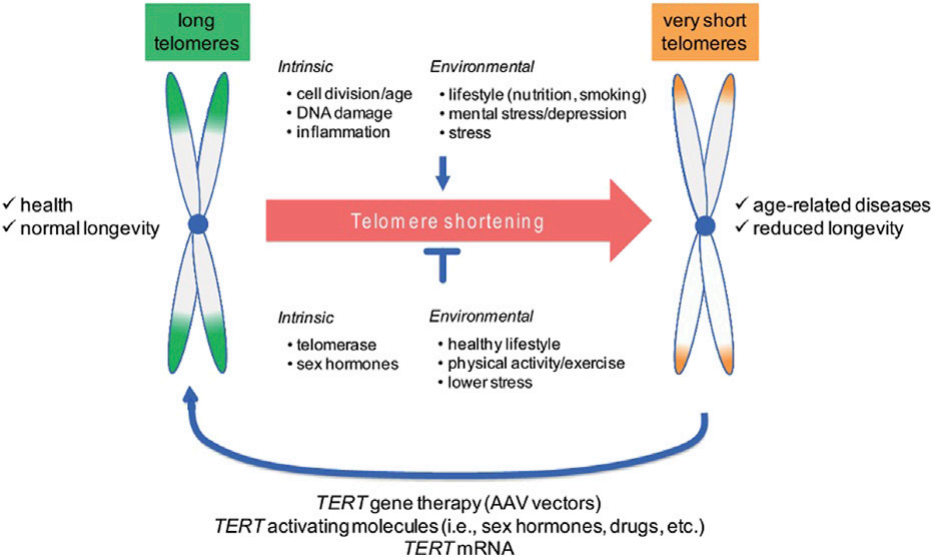
Telomeres in aging and disease (Kirchner et al., 2017). Permission granted by MDPI. Telomere shortening is a life-long process that is influenced by a number of intrinsic and environmental factors that either accelerate or slow down natural telomere attrition, which causes aging and the emergence of age-related diseases. The identification of telomere shortening as a driver of molecular aging has triggered the development of telomerase-based strategies to (re)elongate telomeres and thus to delay aging and associated disease. Abbreviations: AAV, adeno-associated vectors; TERT, telomerase reverse transcriptase. Source: Figure and caption are reproduced from Bär and Blasco (2016a), under the CC BY 4.0 license.

Telomere diseases constitute a growing area of interest in medical research, with several therapeutic interventions being explored. The activation of telomerase enzyme for maintaining telomeres and counter age-related decrease in telomere length can be countered by telomerase gene therapy. The role of telomerase gene therapy in restoring chromosomal stability and reversing tissue degeneration has been demonstrated in animal models including cancer-resistant mice models that exhibited higher telomerase activation in response to therapy, associated with prolongation of lifespan, systematic delay in the process of aging, and integrity of epithelial barriers (Hong and Yun, 2019). TERT gene therapy employs non-integrative adeno-associated vectors and single treatment with the TERT gene is associated with an increase in TERT expression, elongation of telomeres, and extension of lifespan (Figure 3) (Bär and Blasco, 2016b). TERT-based gene therapy may potentially treat human telomere syndromes such as pulmonary fibrosis and aplastic anemia as well as age-related diseases (Bär and Blasco, 2016b; Haycock et al., 2014). Another promising therapeutic approach for optimizing telomere biology is the utilization of small molecules or compounds to restore telomere maintenance in human stem cells (Brenner and Nandakumar, 2020). PAPD5 polymerase functions by destabilizing the RNA component of telomerase, which leads to reduced telomerase activity and mediates the development of human telomere syndromes such as dyskeratosis congenita and pulmonary fibrosis. The administration of a PAPD5 inhibitor, BCH001 or dihydroquinolizinone RG7834, restores the activity of telomerase and the length of telomeres (Nagpal et al., 2020).

The utilization of enzymes including DNA polymerase as a DNA repair enzyme is a promising approach to achieve telomere lengthening (Martinez and Blasco, 2021). The alternative lengthening of telomeres is considered a telomere maintenance mechanism, which is based on a recombination-dependent replication pathway.

ALT-associated PML bodies (APBs) formed due to interaction of SUMOylation (SUMO) with SUMO-interaction motifs (SIM) within promyelocytic leukemia (PML) protein help in scaffold homology-directed telomere lengthening (Zhang et al., 2021). Single stranded region of telomeric DNA with sequence (CCCTAA)n is now established as a biomarker for ALT and can be used as a template for synthesis of telomeric DNA. ALT can be confirmed and quantified in tumor cells by PCR- based detection and quantification of these C-circles (O'Sullivan and Greenberg, 2025). Although the mechanism of formation of C-circles is yet to be elucidated, the involvement of BLM helicase and telomerase lagging strand is reported (Jiang et al., 2024; Lee et al., 2024). In addition, ALT can be detected by other markers such as, APBs, TERRA, G-quadruplexes, along with replication stress response proteins including BLM, FANCM and SLX4. The therapeutic targets of RAD51 dependent ALT pathway are reported to be NBS1, Pol δ, and SMC5/6 while targets for RAD52 dependent pathway include RAD52, SLX4, and POL3/4 (Sohn et al., 2023).

The homologous recombination DNA repair pathways are triggered by the ineffective repair of damaged telomeres. The abnormal chromatin architecture at alternative lengthening of telomeres is susceptible to increased replication stress, mediating the convergence of various gene repair pathways to counter replication stress. Moreover, break-induced synthesis of telomeres involved DNA polymerase δ, initiated by the DNA polymerase η. Damage DNA within alternative lengthening of telomeres triggers both intratelomere and intertelomere recombination, which mediates copying of the telomere template and telomere extension driven by both DNA polymerase δ and DNA polymerase η (Sobinoff and Pickett, 2017). Telomeric RNA is another therapeutic target, which facilitates the elongation of telomeres via recombination-mediated mechanisms. Telomeric RNA accumulates at short telomeres, functioning as an end-capping structure. This allows telomeric RNA to regulate telomeric length, ensuring the maintenance of genome stability. Telomeric RNA is indirectly associated with telomeres, linked through shelterin proteins, and also interacts with various RNA binding proteins. The levels of telomeric RNA tend to be elevated in cells with alternative lengthening of telomeres compared to normal cells. Targeted telomeric double-stranded breaks allow the accumulation of telomeric RNA and the elongation of telomeres (Al-Turki et al., 2024).

Furthermore, advanced techniques such as CRISPR/CAS9 have been employed in activation of RAD51-directed telomeric repair by introducing double stranded breaks in DNA. CRISPR/CAS9 system using fluorescently tagged d-Cas protein can help in telomere repair by upregulating repair systems or reducing cell viability, and increasing telomerase activity and length (Mao et al., 2016). Targeting hTERT promoter leads to upregulation of TERT using CRISPR/CAS9 knock-in. Studies have used CAS9 to target and activate TERT preventing telomere attrition. In addition, spatiotemporal activity of telomerase has also been studied by labelling and imaging using CRISPR/CAS9 (Brane and Tollefsbol, 2019).

**FIGURE 4:**
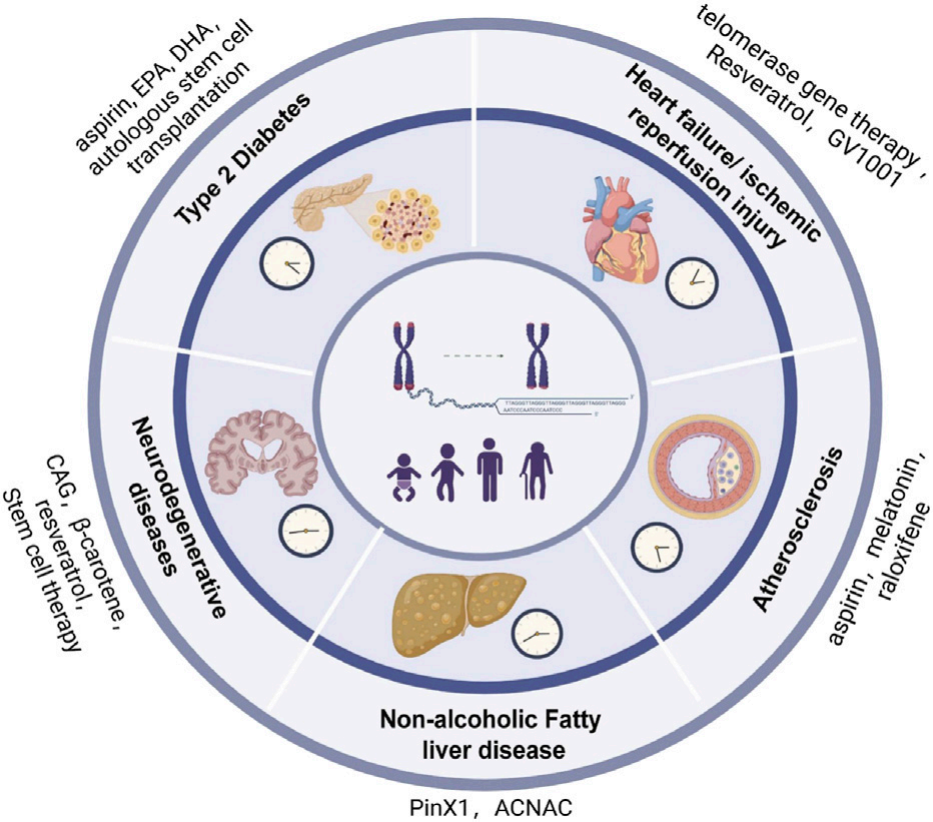
Age-related Chronic Diseases and potential intervention strategies caused by telomere injury. Aspirin, Docosahexaenoic acid (DHA) are used to treat cardiovascular diseases and Type-2 diabetes. These drug increases telomerase activity. Telomerase gene therapy protects cardiomyocytes from apoptosis. Resveratrol inhibits telomerase activity and GV1001 is anti-inflammatory and anti-apotosis drug. These drugs are used to treat heart failure/ischemic reperfusion injury. Stem cell therapy improve brain cell and also protect it from damage. β-caretene and raloxifene regulates telomerase activity. PinX1 and ACNAC increases telomerase activity by inhibiting apoptosis. Melatonin regulates vascular senescence. Source: “Reproduced from Wang et al. (2023). Elsevier Masson SAS. All rights reserved.”

Given the role of reactive oxygen species and oxidative stress in DNA damage, telomere attrition, and premature aging, antioxidant supplementation is a potentially useful approach to mitigate telomere shortening. The administration of antioxidants has been demonstrated to counter telomere shortening and balancing oxidative stress, preventing somatic cells from a decline in their telomere length (Moustakli et al., 2023). Both antioxidants and anti-inflammatory agents have been shown to reduce the rate of telomere attrition during the process of aging. A diet rich in antioxidants is associated with reduced oxidative stress and increased telomeric length. The decrease in the levels of pro-inflammatory cytokines is further linked to a reduction in the rate of telomere attrition (Prasad et al., 2017). Different studies have demonstrated the association between the consumption of a healthy diet, good sleep habits, reduced stress levels, and physical activity with greater telomeric length. Moreover, micronutrients such as specific minerals and vitamins tend to have a protective effect on telomere attrition (Galiè et al., 2020). Anti-inflammatory interventions provide a new therapeutic approach for mediating telomere biology, promoting telomerase activity, and delaying telomere damage. These interventions may include statins, angiotensin-converting enzyme inhibitors, and resveratrol (Figure 4) (Liu et al., 2023).

Traditional Mediterranean diet including healthy foods such as fruits, vegetables, coffee, nuts, wine and foods rich in phytochemicals and antioxidants help in telomere lengthening in contrast to sweetened beverages, meet and processed foods (Galiè et al., 2020). For example, by taking 57 g of pistachios daily for 4 months telomere shortening is significantly prevented in prediabetic patients (Canudas et al., 2019). In contrast, Folate supplementation given to patients with vitamin B12 deficiency leads to cognitive impairment (Mills et al., 2018). In addition, homocystein in access levels also poses drastic affects on brain resulting in stroke, AD, vascular dementia and neural tube defects (Spence, 2019). Anti-inflammatory interventions provide a new therapeutic approach for mediating telomere biology, promoting telomerase activity, and delaying telomere damage (Figure 4).

Although, telomerase can be a good diagnostic and predictive biomarker of human health, its metabolic activity is yet to be elucidated. Telomerase is characterized as a therapeutic taret for many cancers, which shows its complex signalling pathways. Telomerase activation or restoration can somehow lead to activation of cancer cells leading to immortality, which is one of the biggest limitation of this study (Lulkiewicz et al., 2020). Various types of cancers have been associated with telomere length such as basal cell carcinoma, melanoma, glioma, lymphoma, urogenital cancer and lung cancers (Srinivas et al., 2020). Therefore, extensive precautionary research is needed to optimize the telomere therapies to avoid irreversible pathophysiological affects. Various potential risks of telomerase gene therapy may includes various other types of cancer, allergic reaction, damage of organs and tissues, etc. (Okamoto and Seimiya, 2019; Wang et al., 2024; Cacchione et al., 2019).

Telomerase inhibition may affect the formation of blood cells and immune system cells, as well as fertility and wound healing.

## 5 Conclusion

This review has comprehensively explored the association of different molecular and cellular mechanisms of aging with the development of premature aging and age-related metabolic diseases. Telomere attrition or shortening is one of the hallmarks of aging and metabolic diseases, which is implicated in the impairment of genomic stability and cellular functions. The progressive decrease in the length of telomeres is associated with a wide range of age-related pathologies. In addition to telomere shortening, other cellular and molecular mechanisms underlying premature aging include DNA damage, misfolded proteins, epigenetic alterations, cell cycle arrest, stem cell exhaustion, impaired intercellular communication, and aberrant nutrient sensing.

The irreversible pathophysiological process of aging results in the impairment of cellular and tissue functioning, increasing the likelihood of several age-related diseases such as metabolic disorders. Deviation from normal aging, mediated by the aberrant molecular and cellular processes of aging, confers the risk for the emergence of age-related pathologies including metabolic disorders and neurodegenerative conditions. Countering extrinsic and modifiable risk factors such as lifestyle habits and exposure to environmental toxins along with the application of interventions that facilitate the treatment of reversible molecular and cellular changes help promote healthy aging. Stem cell-based therapies that promote stem cell proliferation help prevent stem cell exhaustion, restoring age-associated phenotypes.

The study of telomere biology has revealed potential therapeutic interventions for these disorders. Telomerase gene therapy, small molecule compounds, and DNA repair enzymes are promising approaches to restore telomere maintenance and mitigate the effects of telomere attrition. Additionally, antioxidant supplementation and anti-inflammatory interventions may help prevent telomere shortening and delay the onset of age-related diseases. Further research is needed to fully understand the underlying mechanisms of telomere biology and to develop effective treatments for telomere diseases. While existing research provides evidence supporting the well-established link between telomere attrition and age-related diseases, further studies are required to explore the connection between telomeric length and other age-related and non-age-related conditions. The researcher shall continue to explore novel approaches for telomeric activation, enhanced DNA repair, and strategies for mitigating telomere shortening. Future studies may also evaluate the long-term effects of dietary interventions, exercise, stress management, and other lifestyle factors on telomere length, premature aging, and overall health of humans. Further research needs to develop high-resolution structural understanding, inadequate preclinical models and also try to reduce all potential side effects.

While studies have already emphasized the potential role of telomere length as a biomarker in the field of personalized medicine, it is imperative that the existing knowledge and technology be utilized to tailor preventive measures and treatments accordingly. Moreover, the genetic heritability of telomeres facilitates the development of personalized therapeutic approaches and pharmacogenomic techniques, further supplemented by the genes identified in genome-wide association studies (GWAS) (Gorenjak et al., 2018). Advanced research on telomerase activation may investigate potential therapies that involve safe activation of telomerase, without elevating the risk of cancer as well as explore natural compounds or pharmacological formulations that may delay telomere attrition. It is imperative that stakeholders including policymakers contribute to the development and implementation of public health initiatives as well as advocate for policies that counter environmental stressors, for the common goal of preventing telomere shortening, maintaining telomere length, delaying aging, and countering the development of metabolic disorders.

Maintaining a nutritious, well-balanced diet, regular exercise, cognitive stimulation, stress management, prioritizing restful sleep, building social relationships, getting regular checkups, and addressing modifiable risk factors like blood pressure and cholesterol levels are all part of the strategies for healthy aging and longevity interventions. At the same time, one should adopt an optimistic outlook and partake in activities that make life enjoyable and meaningful.
